# Severe Malnutrition in a Young Adult: Diagnostic Challenges in a Multifactorial Case With Helicobacter pylori Infection

**DOI:** 10.7759/cureus.110623

**Published:** 2026-06-10

**Authors:** Ayubu E Mashambo, Meghna V Solanki, Mandela Makakala, Caroline Ngimba, Masolwa Ngwanasayi

**Affiliations:** 1 Internal Medicine, Aga Khan Hospital Dar es Salaam, Dar es Salaam, TZA; 2 Internal Medicine, Aga Khan University, Dar es Salaam, TZA; 3 Pathology, Aga Khan Hospital Dar es Salaam, Dar es Salaam, TZA; 4 Gastroenterology and Hepatology, Aga Khan Hospital Dar es Salaam, Dar es Salaam, TZA

**Keywords:** chronic infection, helicobacter pylori, malnutrition, micronutrient deficiency, sarcopenia

## Abstract

Severe malnutrition in adults is frequently multifactorial and may result from overlapping nutritional, infectious, inflammatory, and systemic conditions. Identifying the dominant contributors can be challenging, particularly when patients present without classic gastrointestinal symptoms. We report the case of a 24-year-old woman with a more than 10-year history of failure to gain weight despite adequate nutritional intake, associated with progressive bilateral lower limb weakness, anasarca, delayed pubertal development with primary amenorrhea, and severe functional decline. Despite multiple hospitalizations and nutritional interventions, her condition continued to deteriorate. Comprehensive evaluation revealed *Helicobacter pylori *(*H. pylori*)-associated gastritis with micronutrient deficiencies, while further investigation identified a right psoas abscess associated with a marked systemic inflammatory response. Management included *H. pylori *eradication therapy, surgical drainage of the abscess, antibiotic therapy, and structured nutritional rehabilitation. The patient subsequently experienced substantial weight gain, resolution of edema, restoration of mobility, attainment of menarche, and sustained clinical recovery.

This case highlights the multifactorial nature of severe malnutrition and the potential contribution of both chronic gastrointestinal infection and occult deep-seated infection to nutritional decline. In patients with unexplained severe malnutrition accompanied by disproportionate inflammatory markers, clinicians should maintain a high index of suspicion for occult infectious foci and consider early imaging to avoid diagnostic delay and facilitate timely intervention.

## Introduction

Malnutrition in adults is a prevalent yet frequently underdiagnosed clinical condition that significantly contributes to increased morbidity, mortality, prolonged hospitalization, and healthcare expenditure worldwide. It is defined as a state of imbalance between nutritional intake and the body’s physiological requirements, resulting in adverse changes in body composition, impaired physical and cognitive function, and poor clinical outcomes [1]. Importantly, adult malnutrition is increasingly recognized as a disease-associated condition rather than solely a consequence of food deprivation, particularly among hospitalized and chronically ill individuals [2]. The prevalence of malnutrition varies according to age, disease burden, and healthcare setting [3]. Among older adults, malnutrition affects approximately 5-10% of community-dwelling individuals and up to 20-40% of hospitalized patients [4].

Based on underlying mechanisms, malnutrition can be classified into three broad categories: starvation-related malnutrition, disease-related malnutrition without inflammation, and disease-related malnutrition with inflammation. The latter is particularly important in clinical practice because persistent inflammatory activity promotes a catabolic state that accelerates loss of skeletal muscle and functional decline, often limiting the effectiveness of nutritional therapy alone [5].

Chronic infections are a recognized cause of inflammatory malnutrition. Occult deep-seated infections, including intra-abdominal abscesses, osteomyelitis, and infective endocarditis, may present with progressive weight loss and nutritional deterioration even in the absence of prominent localizing symptoms. Sustained inflammation increases metabolic demands and contributes to negative protein balance, resulting in loss of lean body mass and worsening nutritional status. Recognition of these potentially reversible causes is essential when evaluating patients with unexplained malnutrition [6,7].

Malnutrition is associated with a broad spectrum of adverse clinical outcomes, including sarcopenia, impaired wound healing, immune dysfunction, increased susceptibility to infection, delayed recovery from illness or surgery, and neurocognitive impairment related to micronutrient deficiencies [8]. Pathophysiologically, disease-related malnutrition evolves through two interrelated pathways. In the absence of inflammation, reduced intake due to anorexia, dysphagia, or malabsorption predominates. In contrast, inflammatory malnutrition is characterized by cytokine-mediated catabolism of muscle and adipose tissue, compounded by appetite suppression and metabolic inefficiency [1,5].

*Helicobacter pylori *(*H. pylori*)* *infection, while classically associated with peptic ulcer disease and chronic gastritis, can contribute to malnutrition by inducing persistent gastric inflammation, mucosal atrophy, and impaired absorption of micronutrients such as iron, vitamin B12, and folate. Inflammatory mediators triggered by infection may further suppress appetite and promote catabolism [9]. This case report highlights an uncommon but clinically relevant presentation of severe malnutrition in a young adult secondary to *H. pylori* infection and occult infections, underscoring the importance of considering reversible infectious causes in the diagnostic evaluation of unexplained malnutrition.

## Case presentation

A 24-year-old university graduate presented with chronic failure to gain weight for over 12 years, despite reporting adequate intake of balanced meals. Over the preceding five months, she had developed progressive bilateral lower limb edema, hip pain radiating to the thighs, and worsening muscle weakness, culminating in the inability to ambulate. She also reported primary amenorrhea and absence of secondary sexual characteristics. There was no history of gastrointestinal symptoms (such as nausea, vomiting, diarrhea), eating disorders, or behaviors with the intent to lose weight.

She had multiple hospitalizations at different facilities over the years. At one tertiary center, she received nutritional supplementation with plumy nuts, resulting in modest weight gain. She also received several blood transfusions for symptomatic anemia. Despite extensive laboratory and imaging evaluations, no definitive diagnosis had been previously established. Personal and family history were unremarkable.

The patient was oriented, appeared chronically ill, wasted, and pale, with peri-orbital edema and sunken eyes. She had generalized pitting edema. Vital signs revealed borderline hypotension, tachycardia (HR: 125 bpm), tachypnea (RR: 26), and oxygen saturation of 93% on 4 L/min nasal cannula. BMI was 15.8 kg/m². Neurological examination showed marked muscle wasting, reduced tone and strength predominantly in the lower limbs, distal sensory loss, diminished ankle reflexes, and impaired proprioception. Chest examination revealed dullness and decreased air entry in the left lower zone. No lymphadenopathy or hepatosplenomegaly was detected.

Laboratory findings

**Figure 1:**
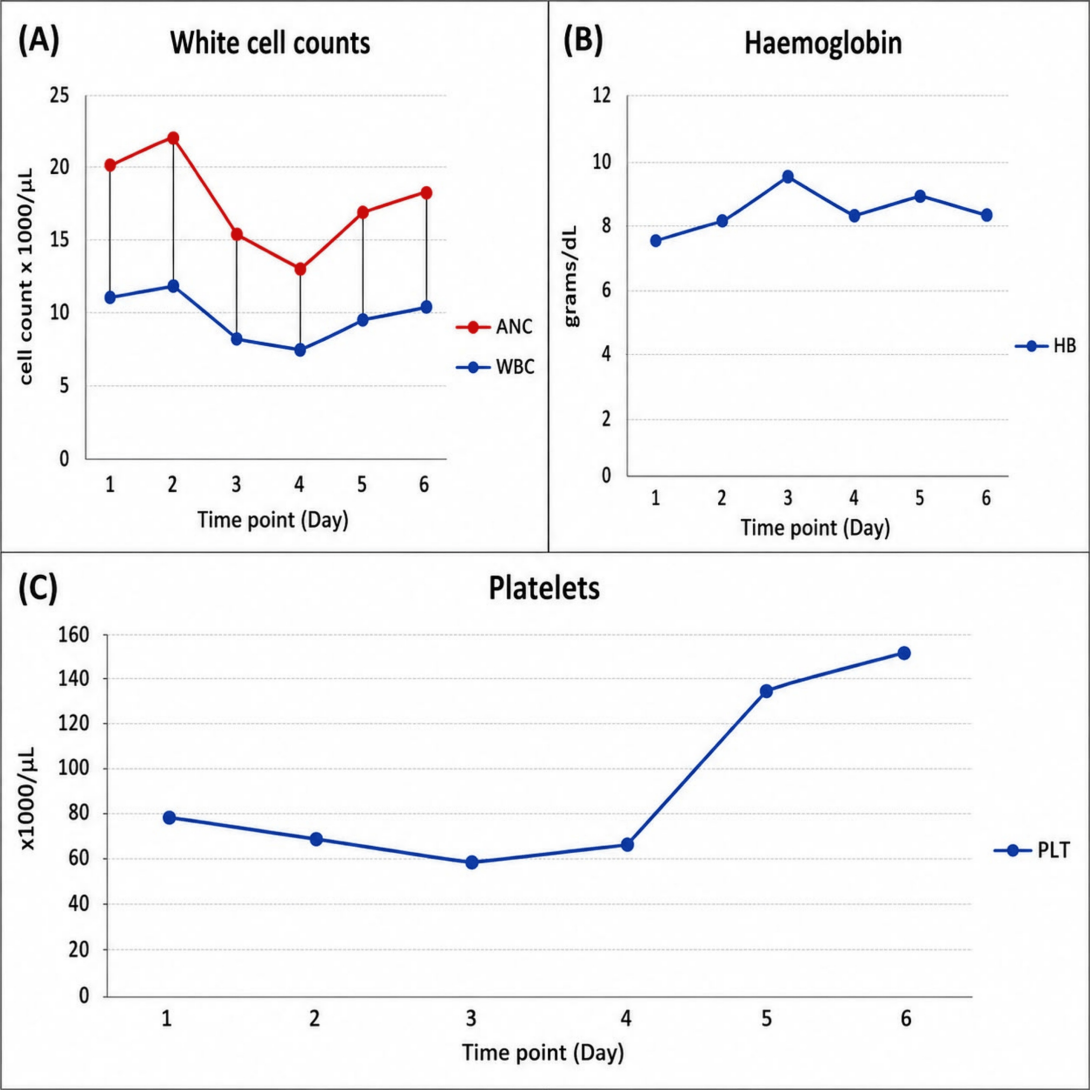
Trends of hematological parameters over time WBC: white blood cell; ANC: absolute neutrophil count; HB: hemoglobin; PLT: platelet A shows that WBC count and ANC fluctuated but remained within expected inflammatory response ranges. B shows that HB showed gradual improvement, correlating with transfusion and nutritional support. C shows PLT initially trended downward, followed by a sharp rise, possibly reflecting resolution of systemic inflammation or recovery from bone marrow suppression

Table 1 and Figure 1 present the patient's laboratory findings over the course of her stay in the ward. 

**Table 1:** Summary of the laboratory findings CRP: C-reactive protein; INR: interquartile range; ANA: antinuclear antibodies; AMA: antimitochondrial antibodies; EMA: anti-endomysial antibodies Baseline laboratory findings showed severe malnutrition with microcytic hypochromic anemia, thrombocytopenia, hypoalbuminemia, profound vitamin D deficiency, elevated inflammatory markers, prolonged INR, and positive fecal occult blood testing. Autoimmune serology was negative, and peripheral smear findings supported nutritional anemia

Investigation	Finding	Reference range
Hemoglobin	3.2-8.2 g/dL	Female: 12-16 g/dL; Male: 13-17 g/dL
Platelet count	68-95 × 10⁹/L	150-450 × 10⁹/L
CRP	60.9 mg/L	<5 mg/L
Procalcitonin	94 ng/mL	<0.05 ng/mL
Vitamin D	3.09 ng/mL	30-100 ng/mL
Albumin	24.6 g/L	35-50 g/L
INR	1.82	0.8-1.2
Peripheral blood smear	Nutritional anemia	Normal smear: normocytic, normochromic red blood cells
Autoimmune panel (ANA, AMA, Anti-TTG, EMA)	Negative	Negative
Urinalysis	No proteinuria or ketonuria	No proteinuria or ketonuria
Stool occult blood test	Positive	Negative

Imaging

**Figure 2:**
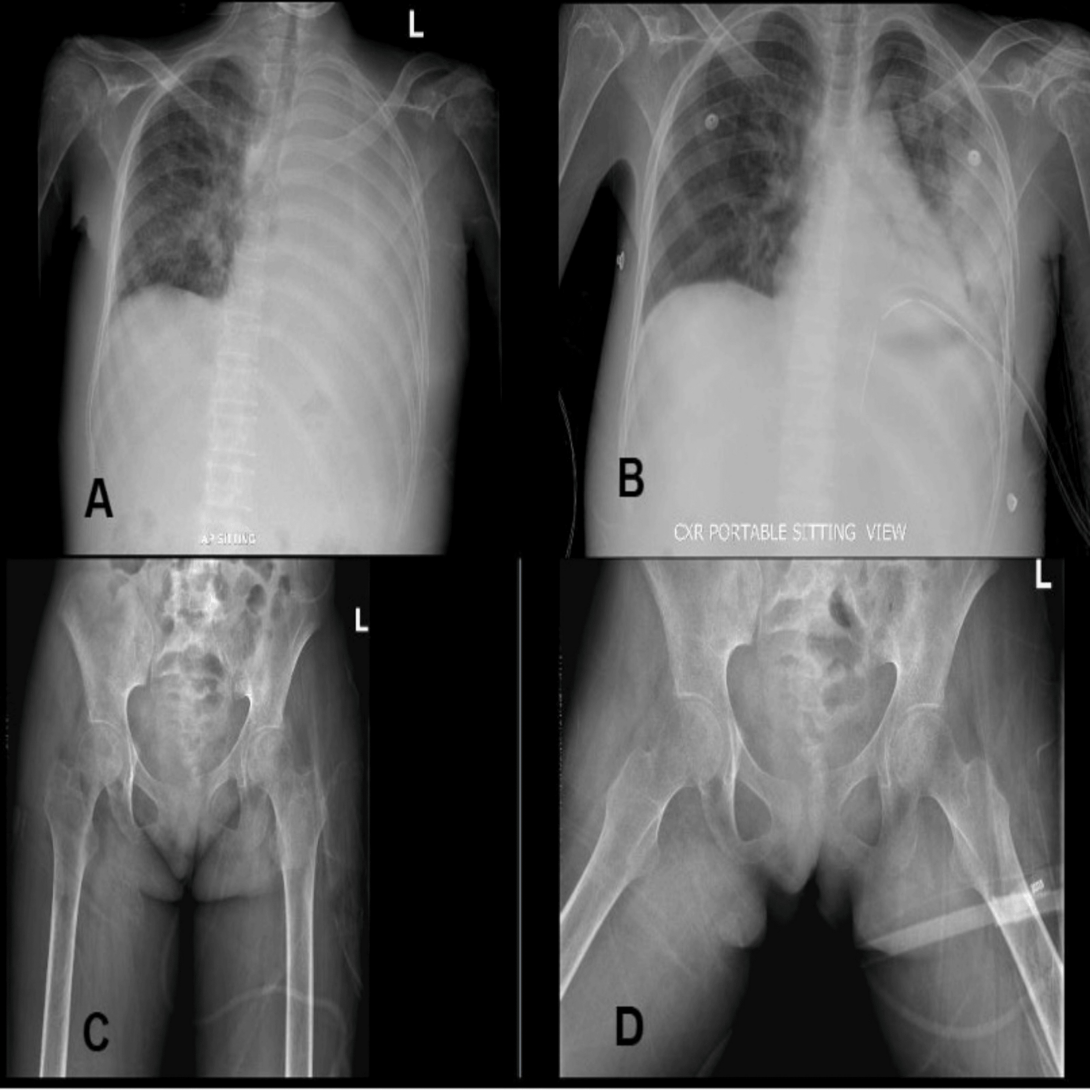
A, Left-sided pleural effusion; B, left-sided pleural effusion after chest tube insertion with reduction; C and D, bilateral hip x-ray showing diffuse osteopenia, no obvious hip joint changes

Chest X-ray revealed a left pleural effusion (Figure 2). Diagnostic chest tube insertion yielded sterile exudative fluid as per the findings listed in Table 2. Musculoskeletal evaluation indicated disuse osteopenia and muscle wasting. An MRI brain and spine ruled out demyelination and compressive pathology.

**Table 2:** Pleural fluid analysis of left-sided pleural effusion AFB: acid-fast bacilli; LDH: lactate dehydrogenase; WBC: white blood cell; RBC: red blood cell; MTB: *Mycobacterium tuberculosis*; RIF: rifampin Based on Light’s criteria, the pleural fluid is classified as an exudate. This conclusion is supported by a pleural fluid protein/serum protein ratio of 0.47 and a pleural fluid LDH/serum LDH ratio of 0.25. Although both ratios fall slightly below the classical cutoff (protein ratio >0.5, LDH ratio >0.6), the absolute pleural fluid protein level of 4.68 g/dL is relatively high, and the clinical context of systemic inflammation and malnutrition raises suspicion for a chronic inflammatory exudate. Additionally, the absence of organisms on Gram stain, Ziehl-Neelsen stain, and culture, alongside a negative GeneXpert MTB/RIF

Parameter	Value	Reference range/expected finding
Pleural LDH (pLDH)	39.38 U/L	Variable; usually < two-thirds upper limit of serum LDH
Pleural protein (pProtein)	4.68 g/dL	Typically <3.0 g/dL in transudates
Pleural WBC (pWBC)	Absent	Normally absent or very few cells
Gram stain	No organism seen	No organisms seen
ZN stain (AFB)	No AFB seen	Negative
Red blood cells (RBC)	Nil	Nil or very few
Gene Xpert MTB/RIF	MTB not detected	MTB not detected
Pleural fluid culture	No bacterial or fungal growth	No growth
Appearance	Pale yellow	Clear/pale yellow
Serum LDH (sLDH)	157 U/L	140-280 U/L
Serum protein (sProtein)	43.8 g/L	60-80 g/L

Endoscopy

**Figure 3:**
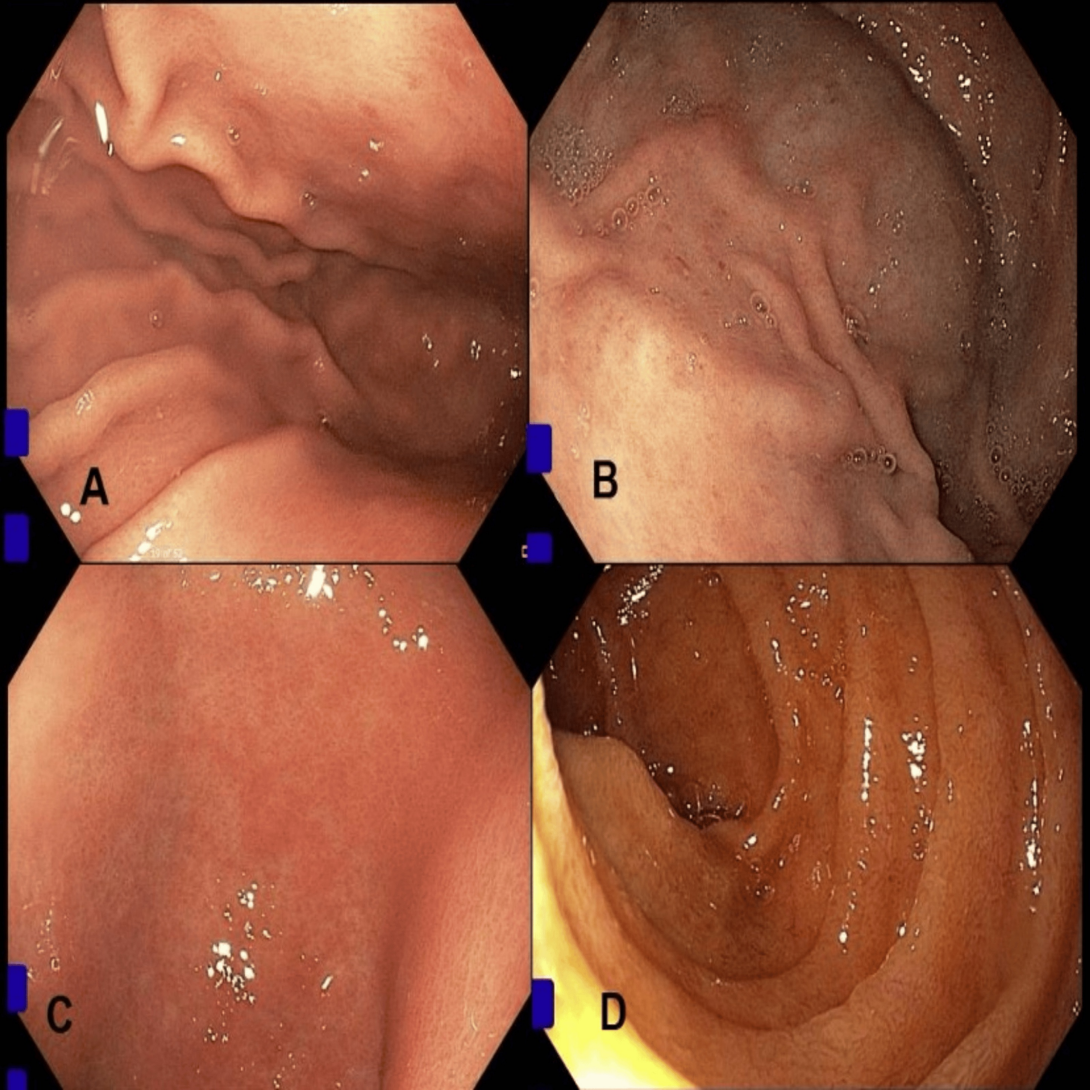
A and B, mild hyperemia and edema of the gastric body; C, mild hyperemia and edema of the gastric antrum; D, normal duodenal mucosa

Upper GI endoscopy showed LA Grade A reflux esophagitis, a 2 cm sliding hiatal hernia, and moderate gastritis involving the antrum and corpus. Duodenal mucosa appeared grossly normal (Figure 3).

Histopathology

**Figure 4:**
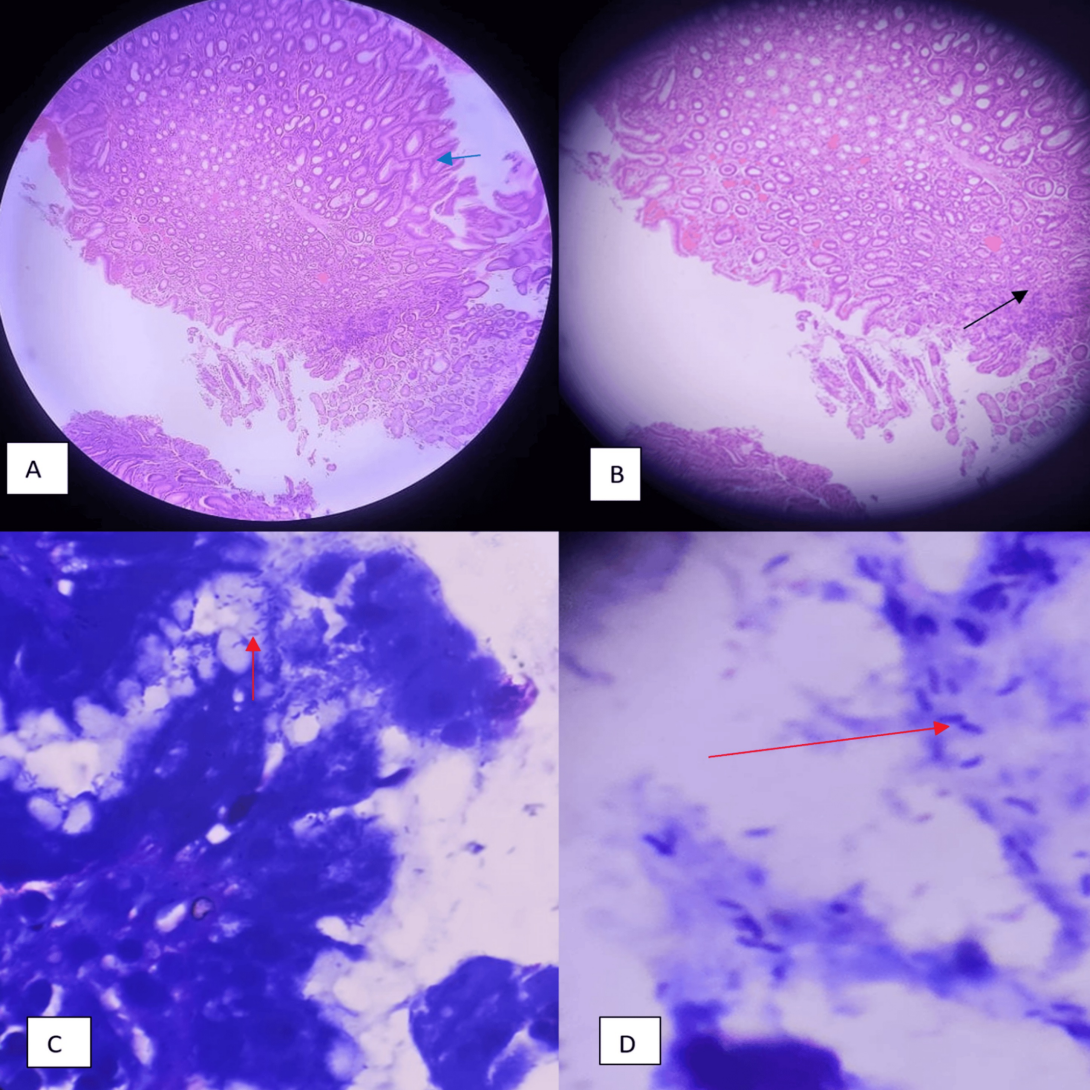
H/E sections A(x10) & B(x20) show fragments of gastric mucosa with active chronic gastritis comprising benign foveolar epithelium with abundant mucin (blue arrow) and inflammatory cells (black arrow). Giemsa stain C & D from the above histology showed moderate H. pylori organisms in the gastric pits (red arrows)

Gastric biopsies revealed chronic active gastritis with *H. pylori *confirmed on special stains. Duodenal biopsies showed chronic duodenitis without villous atrophy, parasitic infection, or granulomas (Figure 4).

The final diagnosis was severe malnutrition according to the Global Leadership Initiative on Malnutrition (GLIM) criteria [10]. The diagnosis was established based on the presence of both phenotypic and etiologic criteria. The phenotypic criteria included significant nonvolitional weight loss, a markedly low body mass index (BMI) of 15.8 kg/m², and reduced muscle mass, which was evident on clinical examination and further supported by radiological evidence of disuse osteopenia. Etiologic criteria included reduced nutrient intake and impaired nutrient assimilation secondary to chronic gastritis, *H. pylori* infection, and possible malabsorption. In addition, the patient demonstrated evidence of a significant inflammatory state, with markedly elevated inflammatory markers including C-reactive protein (CRP) and procalcitonin, as well as a sterile pleural effusion suggestive of ongoing systemic inflammation. Additional diagnoses included *H. pylori*-associated chronic active gastritis, severe iron and vitamin deficiency anemia, peripheral neuropathy, disuse osteopenia, and primary amenorrhea with delayed puberty.

Given the complexity of the patient’s presentation, management required a multidisciplinary approach integrating intensive nutritional rehabilitation with targeted pharmacological therapy. The patient was initially managed in the intensive care unit, where aggressive nutritional rehabilitation was initiated, including high-dose vitamin D, vitamin B12, vitamin K supplementation, intravenous iron sucrose, human albumin infusion, and carefully monitored caloric refeeding. Empiric antimicrobial therapy with ceftriaxone/sulbactam and cotrimoxazole 960 mg every 12 hours to cover possible Whipple’s disease until endoscopic findings with biopsy suggested it was less likely. Supportive management included analgesia and packed red blood cell transfusions for severe anemia. Following confirmation of *H. pylori* infection, eradication therapy using standard triple therapy was commenced and continued on an outpatient basis. Gastric mucosal protection was provided with oral esomeprazole 40 mg twice daily for two months. The patient additionally underwent physical and chest physiotherapy as part of her rehabilitation program.

Two weeks after completion of *H. pylori *eradication therapy, the patient demonstrated marked clinical improvement and nutritional recovery. Her anasarca resolved completely, strength and mobility improved substantially, and her overall well-being markedly increased. She continued regular follow-up in both nutrition and rehabilitation clinics.

Despite these improvements, the patient later continued to report persistent right-sided hip pain, although she remained ambulatory. Magnetic resonance imaging of the hip subsequently revealed a right-sided psoas abscess. She was empirically started on antituberculous therapy; however, she showed no clinical improvement and ultimately discontinued treatment independently. Subsequent re-evaluation led to surgical drainage of the psoas abscess, followed by initiation of broad-spectrum intravenous antibiotics, resulting in significant clinical improvement. Her hip pain resolved, mobility improved further, and she regained functional independence.

At six months of follow-up, the patient demonstrated sustained clinical and nutritional recovery, with a marked improvement in body mass index and functional status. She had resumed work and remained well on regular outpatient review. Importantly, she attained menarche during follow-up, indicating resolution of her primary amenorrhea. This improvement following treatment and nutritional rehabilitation supports the likelihood that her delayed puberty and menstrual dysfunction were consequences of longstanding severe malnutrition and chronic inflammatory disease. 

Timeline of events 

**Figure 5:**
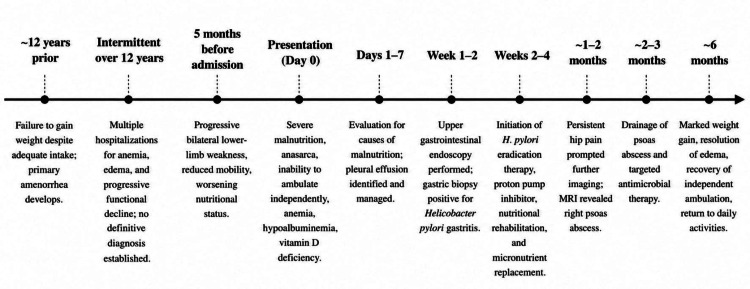
Clinical timeline of presentation, investigations, and management

The sequence of major clinical events, investigations, treatments, and outcomes throughout the patient’s hospitalization and follow-up is summarized in Figure 5. 

## Discussion

Severe malnutrition in this patient was most likely multifactorial, necessitating a broad and systematic diagnostic approach. While *H. pylori* infection was identified, several alternative and coexisting conditions required consideration. Chronic inflammatory disorders, particularly inflammatory bowel disease (IBD) such as Crohn’s disease, remain important differentials given the prolonged disease course, systemic inflammatory features, and subsequent development of a psoas abscess. Although upper gastrointestinal endoscopy did not demonstrate features suggestive of IBD, the absence of colonoscopic evaluation represents a significant limitation in fully excluding this diagnosis.

In addition, the presence of chronic infection and systemic inflammation likely contributed substantially to the patient’s catabolic state. The later identification of a psoas abscess supports the existence of an ongoing inflammatory process, which is a recognized driver of disease-related malnutrition [1].

*H. pylori *infection is known to contribute to iron deficiency anemia and vitamin B12 deficiency through mechanisms involving chronic gastritis, hypochlorhydria, and impaired nutrient absorption [11]. Numerous studies have demonstrated a strong association between* H. pylori* infection and micronutrient deficiencies, particularly in high-prevalence settings [12]. A meta-analysis has further shown reduced serum levels of vitamin B12, folate, vitamin C, and vitamin D among infected individuals [13]. The severe anemia and hypovitaminosis D observed in this patient are consistent with these findings.

Beyond malabsorption, *H. pylori* infection may also influence appetite regulation and metabolic signaling. It has been associated with reduced secretion of ghrelin, an appetite-stimulating hormone, and increased leptin levels, which suppress appetite [14]. Although this patient reported preserved oral intake, her inability to gain weight may reflect altered metabolic signaling and impaired nutrient utilization, rather than inadequate caloric consumption.

However, the severity of malnutrition and systemic manifestations observed in this case are unlikely to be explained by *H. pylori* infection alone. Instead, it is more plausible that *H. pylori *acted as a contributory factor within a broader multifactorial process involving chronic inflammation, infection, and nutritional compromise.

The patient’s nutritional decline was further compounded by the presence of a psoas abscess, which supports a state of ongoing systemic inflammation. Chronic infections are well-recognized to induce a sustained inflammatory response mediated by cytokines such as interleukin-6 (IL-6) and tumor necrosis factor-α (TNF-α), which promote protein catabolism, increase resting energy expenditure, and impair anabolic pathways [15].

In addition, inflammation disrupts micronutrient homeostasis by increasing requirements and impairing utilization. Notably, inflammation-driven iron sequestration through ferritin results in functional iron deficiency, further exacerbating anemia and fatigue. These mechanisms likely contributed to a self-perpetuating cycle of inflammation and malnutrition, amplifying the severity of the patient’s clinical presentation. 

The markedly elevated procalcitonin level in our patient was disproportionate to uncomplicated *H. pylori *gastritis and strongly suggested an underlying severe bacterial infection. In retrospect, the profound inflammatory response, including the sterile pleural effusion, was most likely driven by the occult psoas abscess. The diagnosis was challenging because the patient presented with severe malnutrition, generalized weakness, and musculoskeletal symptoms that could easily be attributed to nutritional complications. Furthermore, psoas abscess is known to present with nonspecific symptoms, and the classical triad of fever, flank pain, and limited hip movement is present in only a minority of patients, often resulting in delayed diagnosis [16]. In our patient, initial orthopedic evaluation and radiographs were unrevealing, while persistent hip pain despite improvement in overall clinical status ultimately prompted an MRI, leading to the diagnosis of psoas abscess.

The patient’s progressive lower limb weakness is consistent with severe disease-related malnutrition, as defined by GLIM criteria [1]. Chronic protein-energy deficiency leads to preferential atrophy of type II muscle fibers, particularly affecting proximal muscle groups and resulting in impaired mobility.

This process was likely compounded by micronutrient deficiencies, including vitamin D deficiency-related myopathy [2], possible vitamin B12-associated neuropathy, and iron deficiency-related fatigue. Together, these factors provide a coherent pathophysiological explanation for the patient’s neuromuscular dysfunction. The patient’s subsequent functional recovery following nutritional rehabilitation and physiotherapy further supports the reversibility of malnutrition-associated neuromuscular impairment.

Chronic malnutrition beginning in adolescence warrants consideration of psychosocial etiologies, including eating disorders such as anorexia nervosa. Although the patient denied restrictive eating behaviors and reported adequate dietary intake, the absence of a structured psychiatric assessment limits the ability to definitively exclude this possibility. Current clinical guidelines emphasize the importance of a multidisciplinary approach, including formal mental health screening, in the evaluation of unexplained malnutrition, particularly in young adults.

The patient’s clinical improvement reflects the cumulative effect of multiple therapeutic interventions, including *H. pylori *eradication, broad-spectrum antimicrobial therapy, nutritional rehabilitation, and surgical drainage of the psoas abscess. Given the simultaneous implementation of these treatments, causal attribution to a single intervention is not possible. This case highlights an important principle in complex clinical medicine: meaningful recovery often results from integrated, multidisciplinary management rather than the resolution of a single etiological factor [15].

Several limitations should be acknowledged. First, a colonoscopic evaluation was not performed, and IBD cannot be definitively excluded. Second, the absence of a formal psychiatric assessment limits the exclusion of underlying eating disorders. Third, the temporal relationship between interventions and clinical improvement precludes definitive conclusions regarding the role of *H. pylori* eradication.

Finally, the presence of a psoas abscess suggests that chronic infection may have been a major driver of the patient’s clinical presentation. The diagnosis of psoas abscess in this patient was challenging because the initial clinical presentation was dominated by severe malnutrition, gastrointestinal symptoms, and generalized functional decline rather than features classically associated with deep-seated retroperitoneal infection. While the patient had localized hip pain and markedly elevated inflammatory markers, early orthopedic evaluation and conventional radiography did not identify a focal musculoskeletal source. Furthermore, clinical improvement during hospitalization, particularly the recovery of lower-limb function, reduced the immediate suspicion for an occult psoas infection. The persistence of hip pain after resolution of the patient's other symptoms ultimately prompted advanced imaging, which revealed the abscess. The psoas abscess was subsequently drained at an external healthcare facility; however, detailed microbiological results, including culture and tuberculosis testing, were not available for review.

## Conclusions

This case highlights the complex and multifactorial nature of severe malnutrition in young adults, where *H. pylori* may act as a significant but not solitary contributor. While *H. pylori*-associated gastrointestinal pathology can lead to micronutrient deficiencies, hormonal dysregulation, and chronic inflammation promoting catabolic wasting, it is unlikely to fully explain profound systemic malnutrition in isolation. In this patient, the coexistence of a soft tissue abscess further amplified the inflammatory burden and contributed to functional iron deficiency, accelerating nutritional decline.

Importantly, meaningful clinical recovery followed a comprehensive strategy incorporating targeted eradication therapy, surgical source control, and structured nutritional rehabilitation. This underscores the necessity of a holistic, multidisciplinary approach when evaluating unexplained malnutrition, with careful avoidance of premature attribution of causality to a single factor. Early recognition and management of reversible infectious and inflammatory contributors are critical to preventing long-term morbidity and restoring functional independence. Furthermore, in cases of unexplained severe malnutrition accompanied by disproportionately high systemic inflammatory markers, clinicians must maintain a high index of suspicion for deep-seated infections and perform targeted early cross-sectional imaging to avoid delayed diagnosis and to prevent premature diagnostic closure on gastrointestinal causes alone.
